# *N*,*N*-Bis(2-pyridylmeth­yl)aniline

**DOI:** 10.1107/S1600536810001881

**Published:** 2010-01-30

**Authors:** Jun-Tao Kang, Guan-Hua Wang, Jing-Wei Xu, Wei Yang

**Affiliations:** a The State Key Laboratory of Electroanalytical Chemistry, Changchun Institute of Applied Chemistry, Chinese Academy of Sciences, Changchun 130022, People’s Republic of China

## Abstract

In the title compound, C_18_H_17_N_3_, the two pyridyl rings make a dihedral angle of 54.55 (13)°. The dihedral angles between the phenyl ring and the two pyridyl rings are 73.61 (13) and 81.40 (13)°. In the crystal, weak inter­molecular C—H⋯π inter­actions are observed.

## Related literature

For bis­(pyridin-2-ylmeth­yl)amine derivatives, see: Komatsu *et al.* (2007 ▶); Royzen *et al.* (2006 ▶); Xiang & Tong (2006 ▶). For related structures, see: Nielsen *et al.* (2005 ▶, 2007 ▶); Bjernemose *et al.* (2003 ▶); Hazell *et al.* (2000 ▶); Ugozzoli *et al.* (2002 ▶). For the synthesis, see: Foxon *et al.* (2007 ▶).
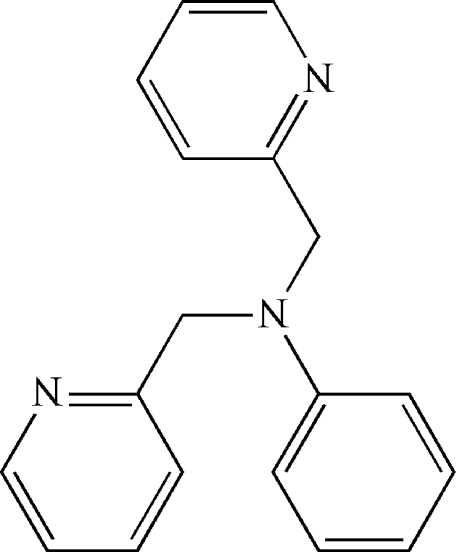


## Experimental

### 

#### Crystal data

C_18_H_17_N_3_
*M* *_r_* = 275.35
Monoclinic, 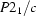

*a* = 11.4866 (19) Å
*b* = 16.811 (3) Å
*c* = 7.7930 (12) Å
β = 101.471 (3)°
*V* = 1474.8 (4) Å^3^
*Z* = 4
Mo *K*α radiation
μ = 0.08 mm^−1^
*T* = 293 K
0.26 × 0.17 × 0.12 mm

#### Data collection

Bruker SMART APEX CCD diffractometer
Absorption correction: multi-scan (*SADABS*; Sheldrick, 1996 ▶) *T*_min_ = 0.981, *T*_max_ = 0.991
7541 measured reflections
2591 independent reflections
1251 reflections with *I* > 2σ(*I*)
*R*_int_ = 0.057

#### Refinement

*R*[*F* ^2^ > 2σ(*F* ^2^)] = 0.049
*wR*(*F* ^2^) = 0.117
*S* = 0.93
2591 reflections
190 parameters
H-atom parameters constrained
Δρ_max_ = 0.14 e Å^−3^
Δρ_min_ = −0.14 e Å^−3^

### 

Data collection: *SMART* (Bruker, 2007 ▶); cell refinement: *SAINT* (Bruker, 2007 ▶); data reduction: *SAINT*; program(s) used to solve structure: *SHELXS97* (Sheldrick, 2008 ▶); program(s) used to refine structure: *SHELXL97* (Sheldrick, 2008 ▶); molecular graphics: *SHELXTL* (Sheldrick, 2008 ▶) and *Mercury* (Macrae *et al.*, 2006 ▶); software used to prepare material for publication: *SHELXTL*.

## Supplementary Material

Crystal structure: contains datablocks global, I. DOI: 10.1107/S1600536810001881/is2511sup1.cif

Structure factors: contains datablocks I. DOI: 10.1107/S1600536810001881/is2511Isup2.hkl

Additional supplementary materials:  crystallographic information; 3D view; checkCIF report

## Figures and Tables

**Table 1:** Hydrogen-bond geometry (Å, °) *Cg*1 and *Cg*2 are the centroids of the C8–C12/N2 and C1–C6 rings, respectively.

*D*—H⋯*A*	*D*—H	H⋯*A*	*D*⋯*A*	*D*—H⋯*A*
C7—H7*A*⋯*Cg*2^i^	0.97	2.98 (4)	3.825 (3)	146
C15—H15⋯*Cg*1^ii^	0.93	2.96 (3)	3.619 (4)	129
C17—H17⋯*Cg*2^iii^	0.93	2.65 (3)	3.530 (3)	159

Symmetry codes: (i) 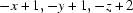; (ii) 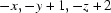; (iii) 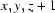.

**Fig. 1.**
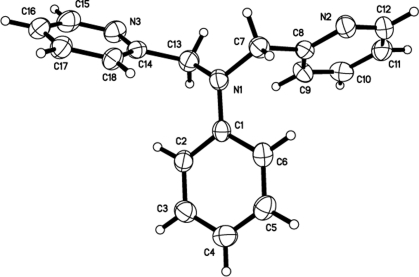
Molecular structure of the title compound. Displacement ellipsoids are drawn at the 30% probability level.

## supplementary crystallographic information

### Comment

Bis(pyridin-2-ylmethyl)amine derivatives are often used as zinc probes (Royzen *et al.*, 2006; Komatsu *et al.*, 2007; Xiang & Tong, 2006). The title compound, *N*,*N*-bis(pyridin-2-ylmethyl)aniline, has been also used as a ligand in metal complexes (Nielsen *et al.*, 2005, 2007; Bjernemose *et al.*, 2003; Hazell *et al.*, 2000; Ugozzoli *et al.*, 2002). Herein, we report the molecular and crystal structure of this compound. The molecule has three rings trending to different orientations, of which the dihedral angle between the two pyridyl rings is 54.55 (13)°, and the dihedral angles between the phenyl ring and the two pyridyl rings are 73.61 (13) and 81.40 (13)°. Intermolecular C—H···π interactions exist in the crystal, which connect molecules into a two-dimensional layer structure.

### Experimental

The title compound was synthesized according to previous reported literature (Foxon *et al.*, 2007). Single crystals suitable for X-ray diffraction were obtained by slow evaporation of a solution of the title compound in acetone at room temperature.

### Refinement

H atoms were placed geometrically with C—H distances of 0.93–0.97 Å and refined using a riding model, with *U*_iso_(H) = 1.2*U*_eq_(C).

### Crystal data

**Table:**

C_18_H_17_N_3_	*F*(000) = 584
*M**_r_* = 275.35	*D*_x_ = 1.240 Mg m^−^^3^
Monoclinic, *P*2_1_/*c*	Mo *K*α radiation, λ = 0.71073 Å
Hall symbol: -P 2ybc	Cell parameters from 649 reflections
*a* = 11.4866 (19) Å	θ = 2.4–19.7°
*b* = 16.811 (3) Å	µ = 0.08 mm^−^^1^
*c* = 7.7930 (12) Å	*T* = 293 K
β = 101.471 (3)°	Block, colorless
*V* = 1474.8 (4) Å^3^	0.26 × 0.17 × 0.12 mm
*Z* = 4	

### Data collection

**Table:**

Bruker SMART APEX CCD diffractometer	2591 independent reflections
Radiation source: sealed tube	1251 reflections with *I* > 2σ(*I*)
graphite	*R*_int_ = 0.057
φ and ω scans	θ_max_ = 25.1°, θ_min_ = 2.2°
Absorption correction: multi-scan (*SADABS*; Sheldrick, 1996)	*h* = −10→13
*T*_min_ = 0.981, *T*_max_ = 0.991	*k* = −19→19
7541 measured reflections	*l* = −9→8

### Refinement

**Table:**

Refinement on *F*^2^	Primary atom site location: structure-invariant direct methods
Least-squares matrix: full	Secondary atom site location: difference Fourier map
*R*[*F*^2^ > 2σ(*F*^2^)] = 0.049	Hydrogen site location: inferred from neighbouring sites
*wR*(*F*^2^) = 0.117	H-atom parameters constrained
*S* = 0.93	*w* = 1/[σ^2^(*F*_o_^2^) + (0.0459*P*)^2^] where *P* = (*F*_o_^2^ + 2*F*_c_^2^)/3
2591 reflections	(Δ/σ)_max_ < 0.001
190 parameters	Δρ_max_ = 0.14 e Å^−^^3^
0 restraints	Δρ_min_ = −0.14 e Å^−^^3^

### Fractional atomic coordinates and isotropic or equivalent isotropic displacement parameters (Å^2^)

**Table:**

	*x*	*y*	*z*	*U*_iso_*/*U*_eq_	
N1	0.27837 (18)	0.47311 (12)	0.8924 (2)	0.0521 (6)	
N2	0.2502 (2)	0.66652 (12)	0.6656 (3)	0.0633 (6)	
N3	0.0590 (2)	0.36999 (13)	1.1010 (3)	0.0649 (6)	
C1	0.3652 (2)	0.42824 (15)	0.8353 (3)	0.0472 (6)	
C2	0.3695 (2)	0.34558 (15)	0.8561 (3)	0.0559 (7)	
H2	0.3130	0.3204	0.9074	0.067*	
C3	0.4559 (3)	0.30090 (16)	0.8019 (3)	0.0664 (8)	
H3	0.4570	0.2461	0.8183	0.080*	
C4	0.5403 (3)	0.33561 (18)	0.7243 (4)	0.0667 (8)	
H4	0.5982	0.3049	0.6877	0.080*	
C5	0.5375 (2)	0.41657 (18)	0.7018 (3)	0.0630 (8)	
H5	0.5941	0.4409	0.6491	0.076*	
C6	0.4515 (2)	0.46266 (16)	0.7564 (3)	0.0553 (7)	
H6	0.4515	0.5175	0.7401	0.066*	
C7	0.2770 (2)	0.55875 (14)	0.8723 (3)	0.0569 (7)	
H7A	0.3572	0.5784	0.9120	0.068*	
H7B	0.2283	0.5811	0.9487	0.068*	
C8	0.2311 (2)	0.58934 (15)	0.6879 (3)	0.0469 (6)	
C9	0.1736 (2)	0.54150 (14)	0.5561 (3)	0.0567 (7)	
H9	0.1621	0.4878	0.5765	0.068*	
C10	0.1332 (2)	0.57366 (17)	0.3930 (3)	0.0648 (8)	
H10	0.0940	0.5420	0.3017	0.078*	
C11	0.1510 (3)	0.65247 (19)	0.3663 (4)	0.0703 (9)	
H11	0.1245	0.6758	0.2573	0.084*	
C12	0.2089 (3)	0.69592 (16)	0.5048 (4)	0.0730 (9)	
H12	0.2207	0.7498	0.4866	0.088*	
C13	0.1675 (2)	0.43629 (15)	0.9124 (3)	0.0588 (7)	
H13A	0.1477	0.3955	0.8233	0.071*	
H13B	0.1055	0.4763	0.8889	0.071*	
C14	0.1647 (2)	0.39913 (13)	1.0875 (3)	0.0470 (7)	
C15	0.0517 (3)	0.33528 (16)	1.2541 (5)	0.0745 (9)	
H15	−0.0216	0.3153	1.2668	0.089*	
C16	0.1452 (3)	0.32745 (16)	1.3924 (4)	0.0713 (9)	
H16	0.1360	0.3019	1.4946	0.086*	
C17	0.2526 (3)	0.35824 (15)	1.3761 (4)	0.0631 (8)	
H17	0.3177	0.3548	1.4683	0.076*	
C18	0.2633 (2)	0.39451 (14)	1.2210 (3)	0.0538 (7)	
H18	0.3357	0.4155	1.2067	0.065*	

### Atomic displacement parameters (Å^2^)

**Table:**

	*U*^11^	*U*^22^	*U*^33^	*U*^12^	*U*^13^	*U*^23^
N1	0.0523 (15)	0.0480 (13)	0.0571 (14)	0.0000 (12)	0.0131 (11)	0.0074 (10)
N2	0.0811 (18)	0.0502 (15)	0.0608 (16)	−0.0035 (12)	0.0198 (13)	0.0024 (11)
N3	0.0521 (16)	0.0707 (16)	0.0754 (18)	−0.0112 (13)	0.0209 (13)	−0.0024 (13)
C1	0.0477 (18)	0.0510 (17)	0.0402 (15)	−0.0018 (14)	0.0025 (12)	0.0056 (12)
C2	0.065 (2)	0.0538 (18)	0.0510 (17)	−0.0028 (15)	0.0166 (14)	0.0048 (13)
C3	0.078 (2)	0.0572 (18)	0.0641 (19)	0.0088 (17)	0.0151 (17)	0.0007 (15)
C4	0.062 (2)	0.075 (2)	0.064 (2)	0.0084 (17)	0.0129 (15)	−0.0038 (15)
C5	0.0474 (19)	0.081 (2)	0.0606 (19)	−0.0089 (17)	0.0098 (14)	0.0056 (16)
C6	0.0490 (17)	0.0583 (17)	0.0554 (17)	−0.0073 (15)	0.0027 (14)	0.0089 (13)
C7	0.067 (2)	0.0516 (17)	0.0518 (17)	0.0031 (14)	0.0115 (14)	0.0003 (12)
C8	0.0492 (17)	0.0454 (16)	0.0465 (16)	0.0047 (13)	0.0104 (12)	0.0013 (13)
C9	0.0641 (19)	0.0460 (15)	0.0554 (18)	0.0047 (14)	0.0007 (14)	0.0014 (14)
C10	0.060 (2)	0.072 (2)	0.0574 (19)	0.0117 (16)	−0.0015 (15)	−0.0101 (15)
C11	0.082 (2)	0.079 (2)	0.053 (2)	0.0183 (18)	0.0200 (17)	0.0141 (17)
C12	0.099 (3)	0.0552 (18)	0.072 (2)	0.0088 (18)	0.0345 (19)	0.0170 (17)
C13	0.0511 (18)	0.0672 (18)	0.0574 (18)	0.0035 (15)	0.0087 (13)	0.0072 (14)
C14	0.0452 (17)	0.0453 (15)	0.0528 (17)	−0.0010 (13)	0.0156 (14)	−0.0046 (12)
C15	0.070 (2)	0.071 (2)	0.094 (3)	−0.0126 (18)	0.043 (2)	−0.0042 (18)
C16	0.091 (3)	0.0649 (19)	0.067 (2)	0.004 (2)	0.037 (2)	0.0013 (16)
C17	0.067 (2)	0.0711 (19)	0.0524 (19)	0.0023 (17)	0.0146 (15)	−0.0002 (14)
C18	0.0471 (18)	0.0613 (18)	0.0550 (18)	−0.0075 (14)	0.0146 (15)	0.0003 (14)

### Geometric parameters (Å, °)

**Table:**

N1—C1	1.393 (3)	C7—H7B	0.9700
N1—C7	1.448 (3)	C8—C9	1.367 (3)
N1—C13	1.452 (3)	C9—C10	1.374 (3)
N2—C8	1.333 (3)	C9—H9	0.9300
N2—C12	1.343 (3)	C10—C11	1.363 (3)
N3—C14	1.332 (3)	C10—H10	0.9300
N3—C15	1.346 (3)	C11—C12	1.362 (4)
C1—C6	1.392 (3)	C11—H11	0.9300
C1—C2	1.399 (3)	C12—H12	0.9300
C2—C3	1.376 (3)	C13—C14	1.507 (3)
C2—H2	0.9300	C13—H13A	0.9700
C3—C4	1.372 (4)	C13—H13B	0.9700
C3—H3	0.9300	C14—C18	1.379 (3)
C4—C5	1.372 (3)	C15—C16	1.368 (4)
C4—H4	0.9300	C15—H15	0.9300
C5—C6	1.388 (3)	C16—C17	1.367 (4)
C5—H5	0.9300	C16—H16	0.9300
C6—H6	0.9300	C17—C18	1.381 (3)
C7—C8	1.519 (3)	C17—H17	0.9300
C7—H7A	0.9700	C18—H18	0.9300
			
C1—N1—C7	119.8 (2)	C8—C9—H9	120.4
C1—N1—C13	120.2 (2)	C10—C9—H9	120.4
C7—N1—C13	116.4 (2)	C11—C10—C9	119.3 (2)
C8—N2—C12	116.3 (2)	C11—C10—H10	120.3
C14—N3—C15	116.6 (2)	C9—C10—H10	120.3
C6—C1—N1	122.3 (2)	C12—C11—C10	117.7 (3)
C6—C1—C2	116.9 (3)	C12—C11—H11	121.1
N1—C1—C2	120.8 (2)	C10—C11—H11	121.1
C3—C2—C1	121.2 (3)	N2—C12—C11	124.6 (3)
C3—C2—H2	119.4	N2—C12—H12	117.7
C1—C2—H2	119.4	C11—C12—H12	117.7
C4—C3—C2	121.3 (3)	N1—C13—C14	116.8 (2)
C4—C3—H3	119.4	N1—C13—H13A	108.1
C2—C3—H3	119.4	C14—C13—H13A	108.1
C3—C4—C5	118.5 (3)	N1—C13—H13B	108.1
C3—C4—H4	120.7	C14—C13—H13B	108.1
C5—C4—H4	120.7	H13A—C13—H13B	107.3
C4—C5—C6	121.0 (3)	N3—C14—C18	123.0 (2)
C4—C5—H5	119.5	N3—C14—C13	114.1 (2)
C6—C5—H5	119.5	C18—C14—C13	122.9 (2)
C5—C6—C1	121.1 (3)	N3—C15—C16	124.2 (3)
C5—C6—H6	119.5	N3—C15—H15	117.9
C1—C6—H6	119.5	C16—C15—H15	117.9
N1—C7—C8	115.68 (19)	C17—C16—C15	118.2 (3)
N1—C7—H7A	108.4	C17—C16—H16	120.9
C8—C7—H7A	108.4	C15—C16—H16	120.9
N1—C7—H7B	108.4	C16—C17—C18	119.2 (3)
C8—C7—H7B	108.4	C16—C17—H17	120.4
H7A—C7—H7B	107.4	C18—C17—H17	120.4
N2—C8—C9	122.8 (2)	C14—C18—C17	118.8 (3)
N2—C8—C7	114.7 (2)	C14—C18—H18	120.6
C9—C8—C7	122.5 (2)	C17—C18—H18	120.6
C8—C9—C10	119.2 (2)		

### Hydrogen-bond geometry (Å, °)

**Table:**

Cg1 and Cg2 are the centroids of the C8–C12/N2 and C1–C6 rings, respectively.

**Table:**

*D*—H···*A*	*D*—H	H···*A*	*D*···*A*	*D*—H···*A*
C7—H7A···Cg2^i^	0.97	2.98 (4)	3.825 (3)	146
C15—H15···Cg1^ii^	0.93	2.96 (3)	3.619 (4)	129
C17—H17···Cg2^iii^	0.93	2.65 (3)	3.530 (3)	159

Symmetry codes: (i) −*x*+1, −*y*+1, −*z*+2; (ii) −*x*, −*y*+1, −*z*+2; (iii) *x*, *y*, *z*+1.
